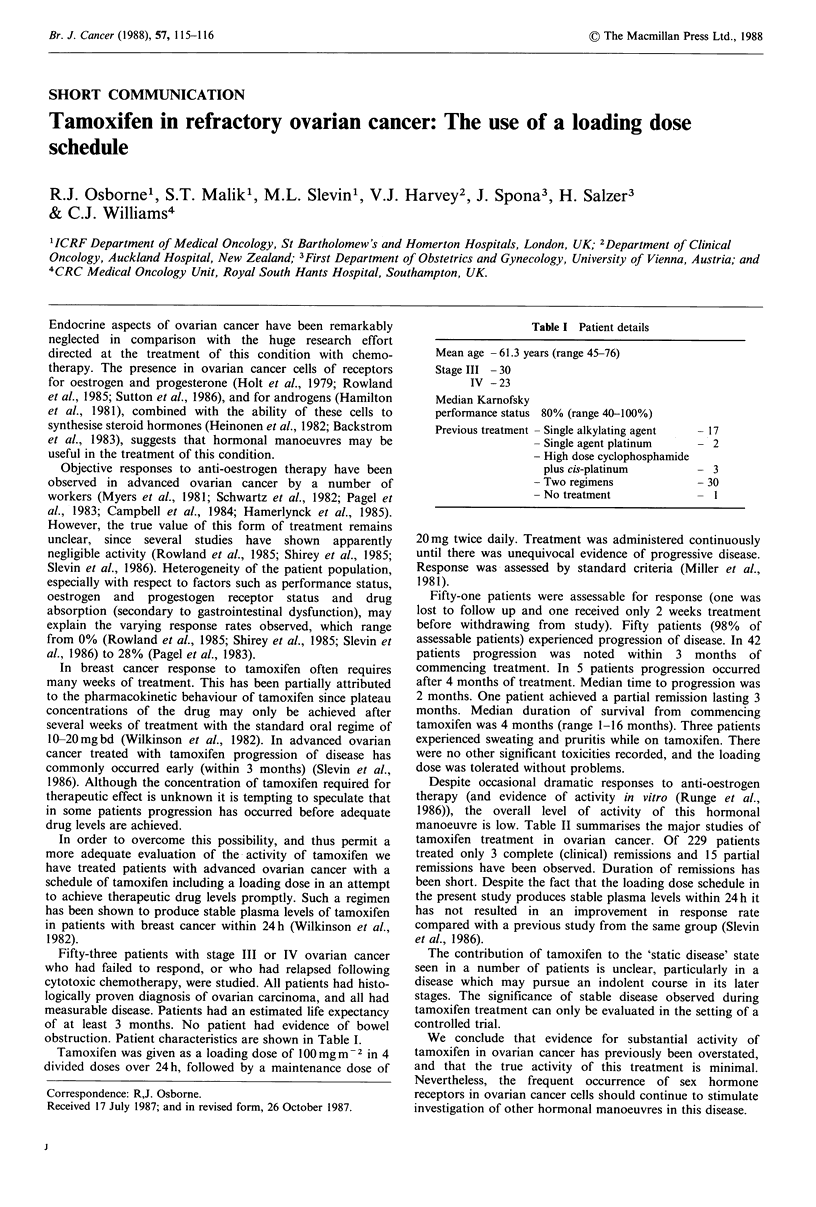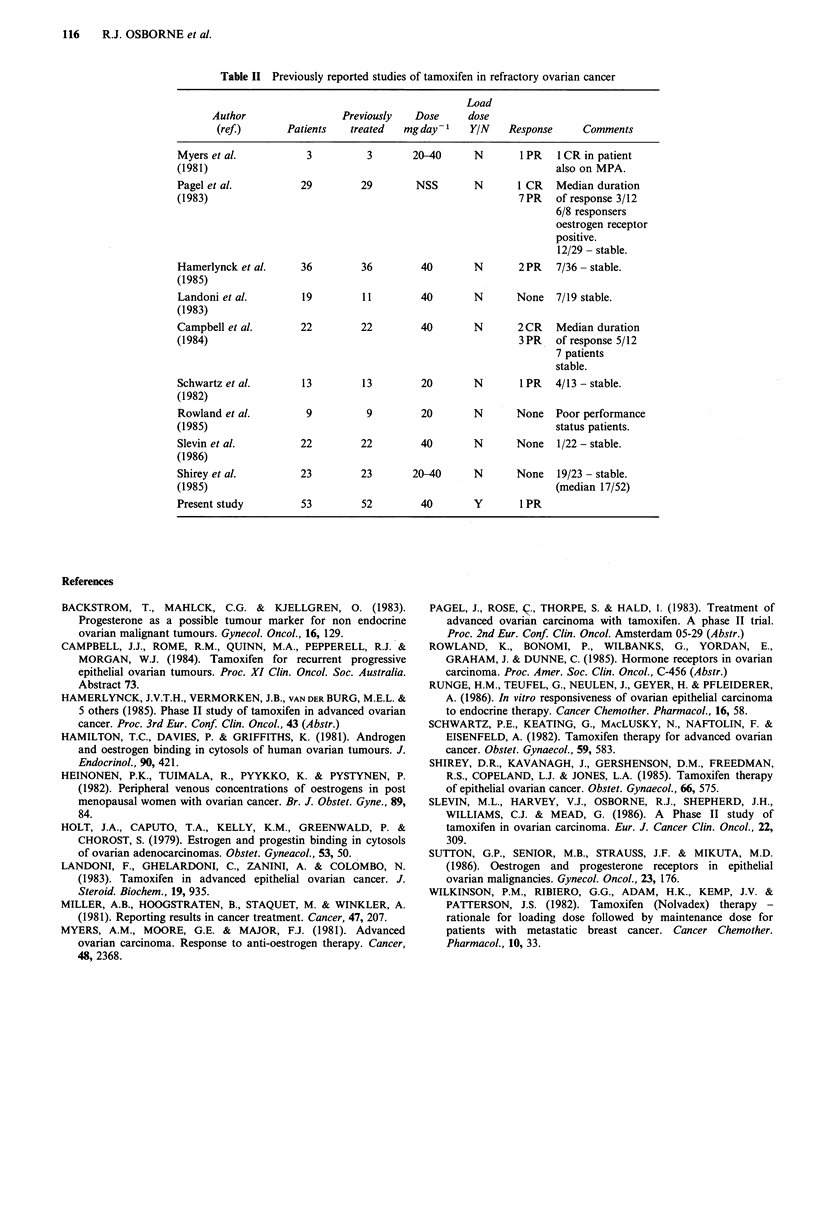# Tamoxifen in refractory ovarian cancer: the use of a loading dose schedule.

**DOI:** 10.1038/bjc.1988.22

**Published:** 1988-01

**Authors:** R. J. Osborne, S. T. Malik, M. L. Slevin, V. J. Harvey, J. Spona, H. Salzer, C. J. Williams

**Affiliations:** ICRF Department of Medical Oncology, St Bartholomew's Hospital, London, UK.


Br. J. Cancer (1988), 57, 115–116 © The Macmillan Press Ltd., 1988

SHORT COMMUNICATION

### Tamoxifen in refractory ovarian cancer: The use of a loading dose schedule

R.J. Osborne[1], S.T. Malik[1], M.L. Slevin[1], V.J. Harvey[2], J. Spona[3], H. Salzer[3] & C.J. Williams[4]

[1]*ICRF Department of Medical Oncology, St Bartholomew's and Homerton Hospitals, London, UK;* [2]*Department of Clinical Oncology, Auckland Hospital, New Zealand;* [3]*First Department of Obstetrics and Gynecology, University of Vienna, Austria; and* [4]*CRC Medical Oncology Unit, Royal South Hants Hospital, Southampton, UK.*

Endocrine aspects of ovarian cancer have been remarkably neglected in comparison with the huge research effort directed at the treatment of this condition with chemotherapy. The presence in ovarian cancer cells of receptors for oestrogen and progesterone (Holt *et al.*, 1979; Rowland *et al.*, 1985; Sutton *et al.*, 1986), and for androgens (Hamilton *et al.*, 1981), combined with the ability of these cells to synthesise steroid hormones (Heinonen *et al.*, 1982; Backstrom *et al.*, 1983), suggests that hormonal manoeuvres may be useful in the treatment of this condition.

Objective responses to anti-oestrogen therapy have been observed in advanced ovarian cancer by a number of workers (Myers *et al.*, 1981; Schwartz *et al.*, 1982; Pagel *et al.*, 1983; Campbell *et al.*, 1984; Hamerlynck *et al.*, 1985). However, the true value of this form of treatment remains unclear, since several studies have shown apparently negligible activity (Rowland *et al.*, 1985; Shirey *et al.*, 1985; Slevin *et al.*, 1986). Heterogeneity of the patient population, especially with respect to factors such as performance status, oestrogen and progestogen receptor status and drug absorption (secondary to gastrointestinal dysfunction), may explain the varying response rates observed, which range from 0% (Rowland *et al.*, 1985; Shirey *et al.*, 1985; Slevin *et al.*, 1986) to 28% (Pagel *et al.*, 1983).

In breast cancer response to tamoxifen often requires many weeks of treatment. This has been partially attributed to the pharmacokinetic behaviour of tamoxifen since plateau concentrations of the drug may only be achieved after several weeks of treatment with the standard oral regime of 10–20 mg bd (Wilkinson *et al.*, 1982). In advanced ovarian cancer treated with tamoxifen progression of disease has commonly occurred early (within 3 months) (Slevin *et al.*, 1986). Although the concentration of tamoxifen required for therapeutic effect is unknown it is tempting to speculate that in some patients progression has occurred before adequate drug levels are achieved.

In order to overcome this possibility, and thus permit a more adequate evaluation of the activity of tamoxifen we have treated patients with advanced ovarian cancer with a schedule of tamoxifen including a loading dose in an attempt to achieve therapeutic drug levels promptly. Such a regimen has been shown to produce stable plasma levels of tamoxifen in patients with breast cancer within 24 h (Wilkinson *et al.*, 1982).

Fifty-three patients with stage III or IV ovarian cancer who had failed to respond, or who had relapsed following cytotoxic chemotherapy, were studied. All patients had histologically proven diagnosis of ovarian carcinoma, and all had measurable disease. Patients had an estimated life expectancy of at least 3 months. No patient had evidence of bowel obstruction. Patient characteristics are shown in Table I.

Tamoxifen was given as a loading dose of $100\,mg\,m^{-2}$ in 4 divided doses over 24 h, followed by a maintenance dose of 20 mg twice daily. Treatment was administered continuously until there was unequivocal evidence of progressive disease. Response was assessed by standard criteria (Miller *et al.*, 1981).

**Table I** Patient details

| | | |
|---|---|---|
| Mean age | – 61.3 years (range 45–76) | |
| Stage III | – 30 | |
| IV | – 23 | |
| Median Karnofsky performance status | 80% (range 40–100%) | |
| Previous treatment | – Single alkylating agent | – 17 |
| | – Single agent platinum | – 2 |
| | – High dose cyclophosphamide plus *cis*-platinum | – 3 |
| | – Two regimens | – 30 |
| | – No treatment | – 1 |

Fifty-one patients were assessable for response (one was lost to follow up and one received only 2 weeks treatment before withdrawing from study). Fifty patients (98% of assessable patients) experienced progression of disease. In 42 patients progression was noted within 3 months of commencing treatment. In 5 patients progression occurred after 4 months of treatment. Median time to progression was 2 months. One patient achieved a partial remission lasting 3 months. Median duration of survival from commencing tamoxifen was 4 months (range 1–16 months). Three patients experienced sweating and pruritis while on tamoxifen. There were no other significant toxicities recorded, and the loading dose was tolerated without problems.

Despite occasional dramatic responses to anti-oestrogen therapy (and evidence of activity *in vitro* (Runge *et al.*, 1986)), the overall level of activity of this hormonal manoeuvre is low. Table II summarises the major studies of tamoxifen treatment in ovarian cancer. Of 229 patients treated only 3 complete (clinical) remissions and 15 partial remissions have been observed. Duration of remissions has been short. Despite the fact that the loading dose schedule in the present study produces stable plasma levels within 24 h it has not resulted in an improvement in response rate compared with a previous study from the same group (Slevin *et al.*, 1986).

The contribution of tamoxifen to the 'static disease' state seen in a number of patients is unclear, particularly in a disease which may pursue an indolent course in its later stages. The significance of stable disease observed during tamoxifen treatment can only be evaluated in the setting of a controlled trial.

We conclude that evidence for substantial activity of tamoxifen in ovarian cancer has previously been overstated, and that the true activity of this treatment is minimal. Nevertheless, the frequent occurrence of sex hormone receptors in ovarian cancer cells should continue to stimulate investigation of other hormonal manoeuvres in this disease.

Correspondence: R,J. Osborne.
Received 17 July 1987; and in revised form, 26 October 1987.

**Table II** Previously reported studies of tamoxifen in refractory ovarian cancer

| *Author (ref.)* | *Patients* | *Previously treated* | *Dose mg day$^{-1}$* | *Load dose Y/N* | *Response* | *Comments* |
|---|---|---|---|---|---|---|
| Myers *et al.* (1981) | 3 | 3 | 20–40 | N | 1 PR | 1 CR in patient also on MPA. |
| Pagel *et al.* (1983) | 29 | 29 | NSS | N | 1 CR 7 PR | Median duration of response 3/12 6/8 responsers oestrogen receptor positive. 12/29 – stable. |
| Hamerlynck *et al.* (1985) | 36 | 36 | 40 | N | 2 PR | 7/36 – stable. |
| Landoni *et al.* (1983) | 19 | 11 | 40 | N | None | 7/19 stable. |
| Campbell *et al.* (1984) | 22 | 22 | 40 | N | 2 CR 3 PR | Median duration of response 5/12 7 patients stable. |
| Schwartz *et al.* (1982) | 13 | 13 | 20 | N | 1 PR | 4/13 – stable. |
| Rowland *et al.* (1985) | 9 | 9 | 20 | N | None | Poor performance status patients. |
| Slevin *et al.* (1986) | 22 | 22 | 40 | N | None | 1/22 – stable. |
| Shirey *et al.* (1985) | 23 | 23 | 20–40 | N | None | 19/23 – stable. (median 17/52) |
| Present study | 53 | 52 | 40 | Y | 1 PR | |

### References

BACKSTROM, T., MAHLCK, C.G. & KJELLGREN, O. (1983). Progesterone as a possible tumour marker for non endocrine ovarian malignant tumours. *Gynecol. Oncol.*, **16**, 129.

CAMPBELL, J.J., ROME, R.M., QUINN, M.A., PEPPERELL, R.J. & MORGAN, W.J. (1984). Tamoxifen for recurrent progressive epithelial ovarian tumours. *Proc. XI Clin. Oncol. Soc. Australia.* Abstract **73**.

HAMERLYNCK, J.V.T.H., VERMORKEN, J.B., VAN DER BURG, M.E.L. & 5 others (1985). Phase II study of tamoxifen in advanced ovarian cancer. *Proc. 3rd Eur. Conf. Clin. Oncol.*, **43** (*Abstr.*)

HAMILTON, T.C., DAVIES, P. & GRIFFITHS, K. (1981). Androgen and oestrogen binding in cytosols of human ovarian tumours. *J. Endocrinol.*, **90**, 421.

HEINONEN, P.K., TUIMALA, R., PYYKKO, K. & PYSTYNEN, P. (1982). Peripheral venous concentrations of oestrogens in post menopausal women with ovarian cancer. *Br. J. Obstet. Gyne.*, **89**, 84.

HOLT, J.A., CAPUTO, T.A., KELLY, K.M., GREENWALD, P. & CHOROST, S. (1979). Estrogen and progestin binding in cytosols of ovarian adenocarcinomas. *Obstet. Gyneacol.*, **53**, 50.

LANDONI, F., GHELARDONI, C., ZANINI, A. & COLOMBO, N. (1983). Tamoxifen in advanced epithelial ovarian cancer. *J. Steroid. Biochem.*, **19**, 935.

MILLER, A.B., HOOGSTRATEN, B., STAQUET, M. & WINKLER, A. (1981). Reporting results in cancer treatment. *Cancer*, **47**, 207.

MYERS, A.M., MOORE, G.E. & MAJOR, F.J. (1981). Advanced ovarian carcinoma. Response to anti-oestrogen therapy. *Cancer*, **48**, 2368.

PAGEL, J., ROSE, C., THORPE, S. & HALD, I. (1983). Treatment of advanced ovarian carcinoma with tamoxifen. A phase II trial. *Proc. 2nd Eur. Conf. Clin. Oncol.* Amsterdam 05-29 (*Abstr.*)

ROWLAND, K., BONOMI, P., WILBANKS, G., YORDAN, E., GRAHAM, J. & DUNNE, C. (1985). Hormone receptors in ovarian carcinoma. *Proc. Amer. Soc. Clin. Oncol.*, C-456 (*Abstr.*)

RUNGE, H.M., TEUFEL, G., NEULEN, J., GEYER, H. & PFLEIDERER, A. (1986). *In vitro* responsiveness of ovarian epithelial carcinoma to endocrine therapy. *Cancer Chemother. Pharmacol.*, **16**, 58.

SCHWARTZ, P.E., KEATING, G., MACLUSKY, N., NAFTOLIN, F. & EISENFELD, A. (1982). Tamoxifen therapy for advanced ovarian cancer. *Obstet. Gynaecol.*, **59**, 583.

SHIREY, D.R., KAVANAGH, J., GERSHENSON, D.M., FREEDMAN, R.S., COPELAND, L.J. & JONES, L.A. (1985). Tamoxifen therapy of epithelial ovarian cancer. *Obstet. Gynaecol.*, **66**, 575.

SLEVIN, M.L., HARVEY, V.J., OSBORNE, R.J., SHEPHERD, J.H., WILLIAMS, C.J. & MEAD, G. (1986). A Phase II study of tamoxifen in ovarian carcinoma. *Eur. J. Cancer Clin. Oncol.*, **22**, 309.

SUTTON, G.P., SENIOR, M.B., STRAUSS, J.F. & MIKUTA, M.D. (1986). Oestrogen and progesterone receptors in epithelial ovarian malignancies. *Gynecol. Oncol.*, **23**, 176.

WILKINSON, P.M., RIBIERO, G.G., ADAM, H.K., KEMP, J.V. & PATTERSON, J.S. (1982). Tamoxifen (Nolvadex) therapy – rationale for loading dose followed by maintenance dose for patients with metastatic breast cancer. *Cancer Chemother. Pharmacol.*, **10**, 33.